# Production of D-lactic acid containing polyhydroxyalkanoate polymers in yeast *Saccharomyces cerevisiae*

**DOI:** 10.1093/jimb/kuab028

**Published:** 2021-04-26

**Authors:** Anna Ylinen, Hannu Maaheimo, Adina Anghelescu-Hakala, Merja Penttilä, Laura Salusjärvi, Mervi Toivari

**Affiliations:** VTT Technical Research Centre of Finland Ltd., P.O. Box 1000, FI-02044 VTT, Espoo, Finland; VTT Technical Research Centre of Finland Ltd., P.O. Box 1000, FI-02044 VTT, Espoo, Finland; VTT Technical Research Centre of Finland Ltd., P.O. Box 1000, FI-02044 VTT, Espoo, Finland; VTT Technical Research Centre of Finland Ltd., P.O. Box 1000, FI-02044 VTT, Espoo, Finland; Department of Bioproducts and Biosystems, School of Chemical Engineering, Aalto University, P.O. Box 11000, FI-00076 Aalto, Espoo, Finland; VTT Technical Research Centre of Finland Ltd., P.O. Box 1000, FI-02044 VTT, Espoo, Finland; VTT Technical Research Centre of Finland Ltd., P.O. Box 1000, FI-02044 VTT, Espoo, Finland

**Keywords:** Poly(D-lactic acid), PHB, Copolymer, Biopolymer, *Saccharomyces cerevisiae*

## Abstract

Polyhydroxyalkanoates (PHAs) provide biodegradable and bio-based alternatives to conventional plastics. Incorporation of 2-hydroxy acid monomers into polymer, in addition to 3-hydroxy acids, offers possibility to tailor the polymer properties. In this study, poly(D-lactic acid) (PDLA) and copolymer P(LA-3HB) were produced and characterized for the first time in the yeast *Saccharomyces cerevisiae*. Expression of engineered PHA synthase PhaC1437_Ps6–19_, propionyl-CoA transferase Pct540Cp, acetyl-CoA acetyltransferase PhaA, and acetoacetyl-CoA reductase PhaB1 resulted in accumulation of 3.6% P(LA-3HB) and expression of engineered enzymes PhaC1Pre and PctMe resulted in accumulation of 0.73% PDLA of the cell dry weight (CDW). According to NMR, P(LA-3HB) contained D-lactic acid repeating sequences. For reference, expression of PhaA, PhaB1, and PHA synthase PhaC1 resulted in accumulation 11% poly(hydroxybutyrate) (PHB) of the CDW. Weight average molecular weights of these polymers were comparable to similar polymers produced by bacterial strains, 24.6, 6.3, and 1 130 kDa for P(LA-3HB), PDLA, and PHB, respectively. The results suggest that yeast, as a robust and acid tolerant industrial production organism, could be suitable for production of 2-hydroxy acid containing PHAs from sugars or from 2-hydroxy acid containing raw materials. Moreover, the wide substrate specificity of PHA synthase enzymes employed increases the possibilities for modifying copolymer properties in yeast in the future.

## Introduction

Global problems related to greenhouse gas emissions and solid waste management have increased interest to replace petroleum-based plastics with the biobased and microbially produced polyhydroxyalkanoate (PHA) polymers. PHAs are biodegradable both in marine (Dilkes-Hoffman et al., 2019) and soil environments (Fernandes et al., 2020), which gives them unique advantage over other plastic materials. Over 150 different hydroxyalkanoate molecules are identified as possible PHA monomers (Koller, 2018; Rehm, 2003). Physical properties of PHA polymers can be upgraded by designing controlled block and random copolymers (McChalicher & Srienc, 2007; Pederson et al., 2006; Yamada et al., 2009). These carry new features in comparison to native PHA polymers and enable the use of PHA materials in even wider range of applications.

The use of 2-hydroxy acids and bioproduction of poly(D-lactic acid) (PDLA) and copolymer P(LA-3HB) have so far been studied mainly in engineered bacterial strains (Choi et al., 2016; Jung et al., 2010; Taguchi et al., 2008; Yang et al., 2010). Interest towards yeast as production host for PHA production has however steadily increased. PDLA production in yeast *Yarrowia lipolytica* was shown with D-lactic acid fed from the culture media (Lajus et al., 2020). In addition, production of poly(hydroxybutyrate) (PHB) and medium chain length (mcl) PHAs from sugars has been demonstrated in engineered yeasts including *Saccharomyces cerevisiae*, *Y. lipolytica*, and *Pichia pastoris* (Gao et al., 2015; Kocharin et al., 2013; Leaf et al., 1996; Li et al., 2016; Poirier et al., 2002). The metabolic engineering of these eukaryotic cells and optimization of their cultivation conditions have already resulted in up to 30-fold increase in PHB and mcl-PHA production, reaching accumulation of 16–30% of polymers of their cell dry weight (CDW) (Carlson & Srienc, 2006; de Las Heras et al., 2016; Portugal-Nunes et al., 2017; Rigouin et al., 2019; Vijayasankaran et al., 2005). Yeasts are attractive hosts for production of PHA polymers, because of their robustness and capability to use cheap raw materials as carbon sources. They also tolerate high concentrations of sugars and organic acids. Yeast bioprocesses are also not prone to phage infections present in bacterial fermentations. The bigger cell size of yeast could potentially enable accumulation of larger PHA granules. Yeast also contain membrane bound organelles, and recently targeting the PHA synthase to peroxisomes increased the PDLA accumulation by over 20-fold in *Y. lipolytica* (Lajus et al., 2020).

**Fig. 1.**
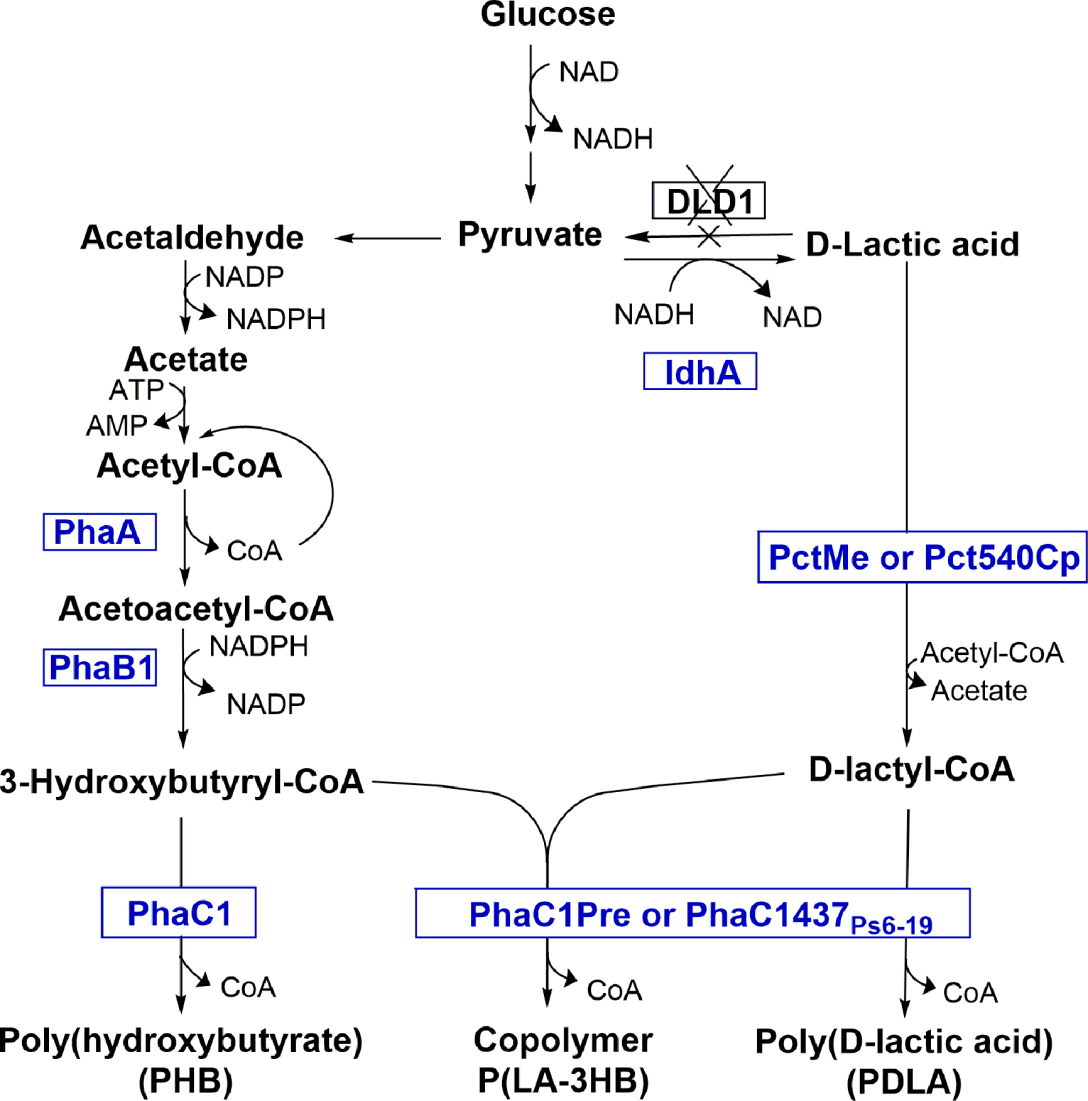
Metabolic pathways for production of poly(hydroxybutyrate) (PHB) (Sandström et al., 2015), poly(D-lactic acid) (PDLA) ( Prabhu et al., 2012; Yang et al., 2010, 2011), and their copolymer P(LA-3HB). D-Lactic acid is produced from pyruvate with a stereospecific D-lactate dehydrogenase (ldhA) from *L. mesenteroides* (Baek et al., 2016).

In the biosynthesis of PDLA, copolymer P(LA-3HB), and other 2-hydroxyacid containing PHA polymers, two key enzymes are needed. Propionyl-CoA transferases (Pct) convert 2-hydroxyacids, including lactic acid, to 2-hydroxyacyl-CoAs, which are then further polymerized by engineered PHA synthases (PhaC) (Fig. 1). Native PHA synthases can polymerize efficiently only 3-, 4-, 5-, and 6-hydroxyacyl-CoA monomers (Choi et al., 2019). Class II PHA synthases from *Pseudomonas* species have been modified to accept also 2-hydroxyacyl-CoAs (Taguchi et al., 2008; Yang et al., 2010). Change in four amino acids at E130, S325, S477, and Q481 enables *in vivo* polymerization of D-lactyl-CoA, glycolyl-CoA, D-2-hydroxybutyryl-CoA, and even some aromatic hydroxyl acids (Lajus et al., 2020; Yang et al., 2018). The ability of these engineered PHA synthases to combine wide range of different monomers provides the possibility to produce polymers with different properties, potentially designed by modeling (Jiang et al., 2020). Since all PHA synthases are strictly stereospecific their polymerization is possible only for R-enantiomers. The production of intracellular D-lactic acid has been enhanced, for example, by expressing a stereospecific D-lactate dehydrogenase (ldhA) and by deleting endogenous D-lactate dehydrogenase (Choi et al., 2016). Several Pct enzymes can convert D-lactic acid to D-lactyl-CoA, for example wild type PctMe from *Megasphaera elsdenii* (Prabhu et al., 2012; Taguchi et al., 2008), and engineered Pct540_Cp_ from *Clostridium propionicum* (Yang et al., 2010)*. Pct540_Cp_* gene has one active mutation at V193A and three non-active mutations at T78C, T669C, and A1125G. For production of PHB, two acetyl-CoA molecules (CoA, Coenzyme A) are converted to acetoacetyl-CoA by acetyltransferase (PhaA). Acetoacetyl-CoA is then further converted to 3-hydroxybutyryl-CoA (3HB-CoA) by acetoacetyl-CoA reductase (PhaB1). Finally 3HB-CoA monomers are combined to long PHB chains by PHA synthase (PhaC1) (Fig. 1).

Although PHA production has been studied extensively in bacterial hosts during the past decades, production in yeast hosts has caught less attention. The main polymer produced has been PHB, and only one recent paper describes the use of 2-hydroxyacid monomers in yeast *Y. lipolytica* (Lajus et al., 2020). We wanted to establish a robust *S. cerevisiae* platform that could potentially use a wide range of monomers. We engineered *S. cerevisiae* strains for production of PDLA, PHB, and their copolymer P(LA-3HB) from glucose. We demonstrated polymerization of 3-hydroxybutyrate and D-lactic acid for co- and homopolymers with wide substrate specificity PHA synthases PhaC1437_Ps6–19_ and PhaC1Pre in *S. cerevisiae*. These enzymes were used since they have shown efficient lactic acid incorporation in P(LA-3HB) copolymer in recombinant *E. coli* (Yang et al., 2011). Formed polymers were extracted and their molecular properties were analyzed. This work paves the way for design and production of new homo- and copolymers in yeast in the future.

## Materials and Methods

### Genes, Plasmids, and Strains

The genes and plasmids used in this study are listed in Table 1. The sequences of PhaA, PhaB1, and PhaC1 were downloaded from GenBank. Genes were codon optimized for expression in *S. cerevisiae*, ordered from Integrated DNA Technologies, and cloned into 2μ plasmids containing strong constitutive promoters by using Gibson Assembly (E2611S, New England BioLabs) or modular cloning method (Lee et al., 2015). *E. coli* TOP10 cells were used as a host for all plasmid constructions. *E. coli* strains were cultivated at 37°C in Luria–Bertani medium with either kanamycin (50 μg ml^–1^) or ampicillin (100 μg ml^–1^).

**Table 1.** Enzymes and Plasmids Used in the Study

Name	Description	Reference
*Enzymes*		
PhaC1437_Ps6–19_	PHA synthase from *Pseudomonas* sp. MBEL 6–19 with amino acid substitutions E130D/S325T/S477G/Q481K, LR699171	Yang et al. (2010)
PhaC1Pre	PHA synthase from *Pseudomonas resinovorans*, with amino acid substitutions E130D/S325T/S477G/Q481K, LR699170	Yang et al. (2011)
Pct540Cp	Propionyl-CoA transferase Pct540 from *Clostridium propionicum*, with V193A and four silent nucleotide mutations T78C, T669C, A1125G, and T1158C, LR699168	Yang et al. (2010)
PctMe	Propionyl-CoA transferase from *Megasphaera elsdenii*, LR699169	Prabhu et al. (2012)
PhaA	Acetyl-CoA acetyltransferase from *Cupriavidus necator*, KP681582	Sandström et al. (2015)
PhaB1	Acetoacetyl-CoA reductase from *C. necator*, KP681583	Sandström et al. (2015)
PhaC1	PHA synthase from *C. necator*, KP681584	Sandström et al. (2015)
LdhA	Stereospecific D-lactate dehydrogenase from *Leuconostoc mesenteroides*, LR699167	Baek et al. (2016)
*Plasmids*		
B9660	*phaA, phaB* and *phaC1* (p*TEF1*-*phaA*-t*ENO1*-p*TDH3*-*phaB*-t*SSA1*-p*PGK1*-*phaC1*-t*ADH1*-*URA3*)	This article
B9663	*pctMe* and *phaC1437_Ps6__–__19_* (p*TEF1*-*pctMe*-t*ENO1*-p*TDH3*-*phaC1437_Ps6__–__19_*-*SSA1*-*LEU2*)	This article
B9664	*pctMe* and *phaC1Pre* (p*TEF1*-*pctMe*-t*ENO1*-p*TDH3*-*phaC1Pre*-t*SSA1*-*LEU2*)	This article
B9665	*pct540Cp* and *phaC1437_Ps6__–__19_* (p*TEF1*-*pct540Cp*-t*ENO1*-p*TDH3*-*phaC1437_Ps6__–__19_*-t*SSA1*-*LEU2*)	This article
B9666	*pct540Cp* and *phaC1Pre* (p*TEF1*-*pct540Cp*-t*ENO1*-p*TDH3*-*phaC1Pre*-t*SSA1*-*LEU2*)	This article
B9667	*pha* and *phaB* (p*TEF1*-*phaA*-t*ENO1*-p*TDH3*-*phaB*-t*SSA1*-*URA3*)	This article

The yeast strains are listed in Table 2. Plasmids were transformed by using lithium acetate method (Gietz & Schiestl, 2007). Strains H3900 (CEN.PK102-5B) and H3895 (CEN.PK113-5D), kindly provided by Dr. P. Kötter (Institut für Mikrobiologie, J.W. Goethe Universität Frankfurt, Germany), were used as parental strains. The D-lactate dehydrogenase gene (*DLD1*) was deleted to increase availability of D-lactic acid *in vivo* (Baek et al., 2016) by integration of stereospecific D-lactate dehydrogenase gene (*ldhA*) from *Leuconostoc mesenteroides* under the *TDH3* promoter. *PhaA* and *phaB1* genes with *TEF1* and *TDH3* promoters, respectively, were integrated into the *his3-Δ1* locus.

**Table 2.** Yeast Strains Used in the Study

Name	Description	Product
H3900	*S. cerevisiae*, CEN.PK102–5B (*MATa his3-Δ1 ura3–52 leu2–3 112 TRP1 MAL2–8c SUC2*)	
H3895	*S. cerevisiae*, CEN.PK113–5D (*MATa HIS3 ura3–52 LEU2, TRP1 MAL2–8c SUC2*)	
H5513	H3900 with integration of *ldhA* into *DLD1* locus (*MATa his3-Δ1 ura3–52 leu2–3 112 TRP1 MAL2–8c SUC2, DLD1::ldhA*)	D-Lactic acid
H5514	H5513 with integration of *phaA, phaB* and *HIS3* to *his3-Δ1* locus (*MATa HIS3 ura3–52 leu2–3112 TRP1 MAL2–8c SUC2*, *DLD1::ldhA, PhaA, PhaB*)	D-Lactic acid, 3-HB-CoA
H5519	H5513 with B9663	PDLA
H5520	H5513 with B9664	PDLA
H5521	H5513 with B9665	PDLA
H5522	H5513 with B9666	PDLA
H5523	H5514 with B9665	P(LA-co-3HB)
H5524	H5514 with B9666	P(LA-co-3HB)
H5529	H3895 with B9660	PHB
H5531	H3895 with B9667	3HB-CoA

### Shake Flask Cultivations

Yeast strains were cultured in synthetic complete (SC) media lacking either leucine (strains H5519–H5524) or uracil (strains H5529 and H5531). Control strains without plasmids (H5513, H5514) were grown in SC medium. All cultivations were supplemented with 20 g l^–1^ glucose as a carbon source and carried out in 250 ml Erlenmeyer flasks at 30°C with 220 rpm shaking. Inocula were grown in same liquid media and washed twice with sterile distilled water before starting the cultivations in 50 ml of fresh growth media for 72 hr. In the first round, three different biological replicates of each strain were grown and analyzed for their PHA production. Cultivation and analysis of the best producing replicate of each strain was then repeated with three identical replicates, starting from OD_600_ 5 and from OD_600_ 0.2.

### Cell Growth, pH, and Extracellular Metabolites

The cell growth was monitored by measuring the optical density (OD_600_) of the cultures at 600 nm with VitroSpec 2100 Pro (Amersham Biosciences) and by measuring the CDW from filtered samples. Before the CDW analysis, glass microfiber filters (55 mm, GF/B Whatman) were dried over night at 100°C and weighted. For analysis, samples of 2 ml were filtered, washed three times, dried at 100°C overnight, and weighted. The CDW values of PHB producing strains were calculated based on conversion of measured OD_600_ values to CDW, since these growth parameters showed linear correlation with *R*^2^ value of 0.98 in the OD_600_ range 0.99–21.35 (unpublished). The pH of the cultivation media was measured with Innolab pH 720 instrument (WTW) and Sentix Mic electrode (WTW). Extracellular metabolites and media components (glucose, ethanol, acetate, glycerol, and lactic acid) were analyzed with high-performance liquid chromatography (HPLC) (Toivari et al., 2010) using 5 mM sulfuric acid as eluent.

### Polymer Quantitation from Lyophilized Cells with GC–MS

Gas chromatography mass spectrometry (GC–MS) was used to verify polymer compositions and to quantify the amount of polymer produced by each strain in relation to their CDW. Cells were collected by centrifugation at 4000 rpm for 6 min, washed three times with water, and lyophilized overnight. Ten milligrams of each sample were subjected to methanolysis for 140 min at 100°C water bath in a solution containing 1 ml chloroform, 20 μl internal standard (3-hydroxybutyric acid), 150 μl sulfuric acid, and 830 μl methanol. Samples were cooled to room temperature and water-soluble particles were removed by addition of 0.5 ml of distilled water. Chloroform phase was analyzed by using gas chromatography system (7890, Agilent) and HP-FFAP column (19091F-102, Agilent). PLA, l-lactic acid, and 3-hydroxybutyric acid were used as standards.

### Polymer Extraction

Polymers were extracted from 600 to 2000 mg of lyophilized cells with chloroform. Lyophilized cell pellets were grinded with metallic spoon, divided into 140–300 mg aliquots, supplemented with 5 ml of chloroform in 12 ml glass screw cap tubes, and boiled in 95°C water bath for 3 hr. The solutions were stirred over night at room temperature, filtered with 0.45 μm PTFE filters, and concentrated to 300 μl. Twenty-fold volume of ice-cold methanol was added to all samples and tubes were centrifuged at 3000 rpm for 20 min. Supernatants were removed. In addition to methanol washing, PDLA and P(LA-3HB) samples for size exclusion chromatography (SEC) analysis were washed also with diethyl ether. PDLA and P(LA-3HB) were dissolved in 100 μl chloroform and 20-fold ice-cold diethyl ether was added to the samples, followed by centrifugation at 3000 rpm for 20 min and removal of supernatant. Remaining precipitated polymers were dried and weighed.

### Analysis of the Purified Polymers

Nuclear magnetic resonance (NMR) analysis was used to verify the monomer compositions and polymer structures. PDLA, PHB, and P(LA-3HB) polymers were dissolved to final concentration of 830 mg l^–1^ in 600 μl chloroform containing 0.03% (v/v) tetramethylsilane standard. Each polymer was analyzed in three replicates in 5 mm NMR tubes. The NMR spectra were recorded with a 600 MHz NMR spectrometer (Avance III, Bruker) equipped with an inverse detection QCI H-P/C/N-D cryoprobe. The polymer samples were measured at 22°C without solvent suppression. The repetition rate for the 1D ^1^H was 4.6 s. The zero quantum filtered total correlation spectroscopy (TOCSY) spectra were recorded using Bruker's pulse program *dipsi2gpphzs* with 120 ms DIPSI2 spinlock. The phase sensitive, multiplicity edited heteronuclear single quantum correlation (HSQC) spectra were recorded using Bruker's pulse program *hsqcedetgpsisp2.2*, which uses Echo/Antiecho-TPPI selection and shaped pulses (CHIRP) for all 180° pulses. ^13^C decoupling during acquisition was achieved by adiabatic CHIRP decoupling. The one-bond ^13^C–^1^H coupling constant was set to 150 Hz (1/4 *J* = 1.6667 ms) and the relaxation delay was 1 s. For TOCSY and HSQC experiments, a matrix of 2048 × 256 data points was collected, zero-filled once in F1 and a *π*/2 shifted squared sine-bell weighting function was applied in both dimensions prior to the Fourier transformation. The absolute value heteronuclear multiple bond correlation (HMBC) spectra were recorded without decoupling using Bruker's pulse program *hmbcgpndqf*. The one-bond ^13^C–^1^H coupling constant was set to 150 Hz and the long-range ^13^C–^1^H coupling constant was set to 8 Hz (1/2 *J* = 3.3333 ms). A matrix of 4096 × 256 points was collected, zero-filled with linear prediction to 4096 × 1024 points and an unshifted sine-bell weighting function was applied in both dimensions prior to the Fourier transformation. All spectra were processed on Topspin 3.5 pl 5 software (Bruker).

The molecular weight measurements were performed with SEC. Three replicates of the PHB sample and a commercial PHB standard (363502, Sigma) were dissolved in 1,1,3,3,3-hexafluoro-2-propanol (HFIP) for 3 days. The PDLA and P(LA-3HB) samples, grown from starting OD_600_ of 0.2, and a commercial PHB standard (363502, Sigma) were dissolved in chloroform and mixed in room temperature for 7 days. At the fourth day, chloroform samples were heated to 80°C for 30 min to ensure complete dissolution. All samples were filtrated with 0.45 μm syringe filters before the analysis. The HFIP eluent with 5 mM sodium trifluoroacetate was delivered at a rate of 0.5 ml min^−1^ at 40°C and chloroform eluent at a rate of 0.6 ml min^−1^ at 30°C. The HFIP system was equipped with Waters styragel HR-4E column and HR 5 column and the chloroform system with styragel HR 4 and 3 columns. All systems contained a pre-column and a refractive index detector (2414, Waters). The molecular weights were calculated either against 10 polystyrene standards of 1260–3,040,000 g mol^−1^ in chloroform or against nine poly(methyl methacrylate) standards of 2710–1,667,000 g mol^−1^ (Agilent) in HFIP, using 3rd order fit (*R*^2 ^= 0.998–0.999) and Waters Empower 3 software.

Differential scanning calorimetry (DSC) analysis was used to determine the polymer melting temperatures. Differential scanning calorimeter (DSC2, Mettler Toledo) was equipped with intra-cooler (TC100MT, Huber) and run under nitrogen atmosphere. Samples of 3–6 mg were weighed into 40 μl aluminum crucibles, lids were pricked, and crucibles were closed with cold-pressing. Samples were heated twice with heating rate 10°C min^–1^. In the first scan, the samples were cooled down to −60°C and heated to 75°C. In the second scan, samples were cooled down to −60°C and heated to 250°C. Results were obtained with Mettler Toledo STARe software (version 13.0). The data were collected from the second heating scan. PHB samples were analyzed in two replicates.

GC–MS analysis of the purified polymers was performed to complement the corresponding NMR results and GC–MS results from the lyophilized cells. Samples of 0.5 mg were subjected to methanolysis and analyzed in the same way as lyophilized cells. Each polymer was analyzed in three replicates.

## Results

### PDLA, P(LA-3HB), and PHB Producing *S. cerevisiae* Strains

*S. cerevisiae* strains containing PDLA, P(LA-3HB), or PHB pathways, presented in Fig. 1, were constructed. To demonstrate the polymer production, three transformants from each strain (H5519–H5524, H5529) were grown on media with 20 g l^–1^ glucose as a carbon source, starting from OD_600_ 2. Cells were collected after 72 hr, washed, lyophilized, and the polymer content was analyzed with GC–MS method. All strains expressing ldhA, Pct, and PhaC (H5519–H5522) enzymes produced PDLA. The control strain (H5513) lacking the PHA synthase did not show any lactic acid in the GC–MS analysis, even though extracellular D-lactic acid concentration at 72 hr was 7.44 ± 0.86 g l^–1^. This indicates that either the intracellular concentration of free D-lactic acid was very low or that free D-lactic acids was efficiently removed from the cells during the washing prior to GC–MS analysis. Result demonstrates that free lactic acid was not distorting the polymer accumulation results in GC–MS analysis. Polymer content of PDLA producing strains varied between the different transformants. This variation was as high as variation between strains expressing different PHA synthases. The highest achieved PDLA content was 0.88% of CDW with strain H5520 expressing PctMe and PhaC1Pre. The P(LA-3HB) copolymer producing strains (H5523 and H5524) were grown and analyzed similarly to the PDLA-producing strains. Both strains produced the copolymer, the polymer content being higher in the strain H5523 expressing Pct540Cp and PhaC1437_Ps6–19_. The highest detected content was 3.07% polymer of CDW, containing 1.97% D-lactic acid and 1.12% 3-hydroxybutyric acid. As a comparison, the PHB producing strains (H5529) produced 6.36– 9.44% polymer of CDW. The transformants that accumulated the highest polymer quantities were chosen for further analyses.

### Characterization of PDLA, P(LA-3HB), and PHB Produced in *S. cerevisiae*

**Fig. 2.**
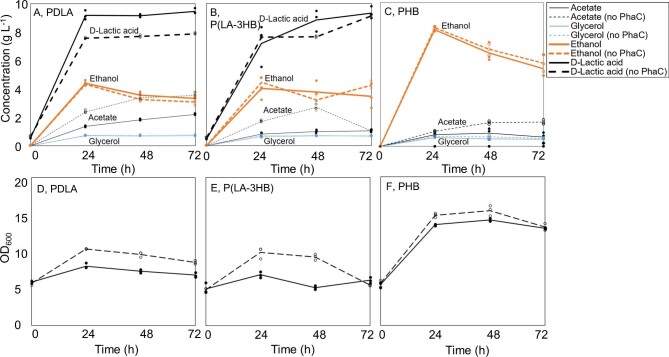
Results from shake flask experiment starting from OD_600_ 5. (A–C) D-Lactic acid, ethanol, acetate, and glycerol concentrations (g l^–1^) in the culture media. (D–F) Cell growth as OD_600_. The lines represent average results from three replicates and the circles individual data points. Polymer strains are marked with continuous lines and their control strains with dashed lines. Strains: A: PDLA: strain producing poly(D-lactic acid) (H5520), D-LA: strain producing D-lactic acid (H5513); B: P(LA-3HB): strain producing copolymer of lactic and 3-hydroxybutyric acids (H5523), D-LA and 3HB-CoA: strain with D-lactic acid and 3-hydroxybutyryl-CoA pathways (H5514); C: PHB: strain producing poly(hydroxybutyrate) (H5529), 3HB-CoA: strain with 3-hydroxybutyryl-CoA pathway (H5531). PhaC: PHA synthase.

**Fig. 3.**
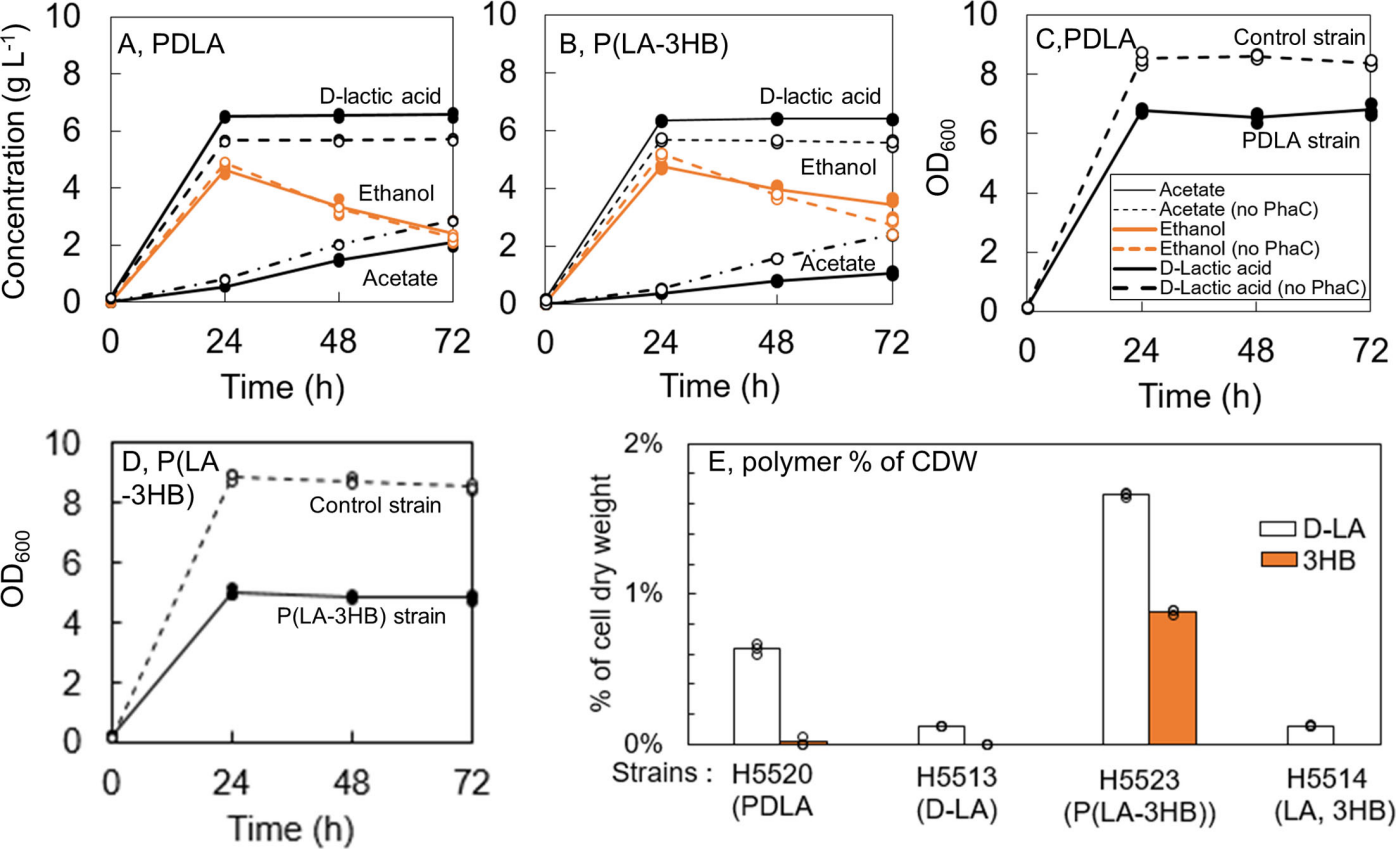
Results from cultivation of PDLA (H5520) and P(LA-3HB) (H5523) strains in shake flasks with starting OD_600_ 0.2. (A, B) D-Lactic acid, ethanol, and acetate concentrations (g l^–1^) in the culture media. (C, D) Cell growth as OD_600_. (E) Monomer composition of *S. cerevisiae* strains [% cell dry weight (CDW) of lyophilized cells]. Data points from each replicate are marked with circles.

The transformants of best polymer producing strains (H5520, H5523, and H5529), and their corresponding control strains, were grown in three parallel cultivations starting from OD_600_ 5 (Fig. 2). In addition, the PDLA and P(LA-3HB) producing strains were grown in three parallel cultivations from starting OD_600_ 0.2 to repeat the experiment and to produce PDLA and P(LA-3HB) for SEC analysis (Fig. 3). Cell growth, extracellular metabolite production, carbon source consumption, and pH were measured every 24 hr. When cultivation started from OD_600_ 5, glucose was entirely consumed in all cultivations during the first 24 hr. When cultivation started from OD_600_ 0.2, small residual glucose concentrations of 0.06 and 0.36 g l^–1^ were measured from the media at 24 hr with PDLA and P(LA-3HB) strains, respectively. All control strains grew better than the polymer producing strains. Cultures did not grow or grew only very little after 24 hr when glucose was depleted. Cultivation was however continued for additional 48 hr to ensure complete polymerization of available monomers. Strains expressing *ldhA* gene produced 5.6–9.5 g l^–1^ D-lactic acid and approximately 50% less ethanol compared to strains without *ldhA*. Strains able to produce PHB and P(LA-3HB) produced less acetate compared to strain producing PDLA. With D-lactic acid producing strains, the pH decreased from 5.5 to 2.6 (± 0.03) during the first 24 hr, whereas the pH of strains with PHB and 3HB-CoA pathways (H5529 and H5531) decreased only to 3.24 (±0.10).

**Fig. 4.**
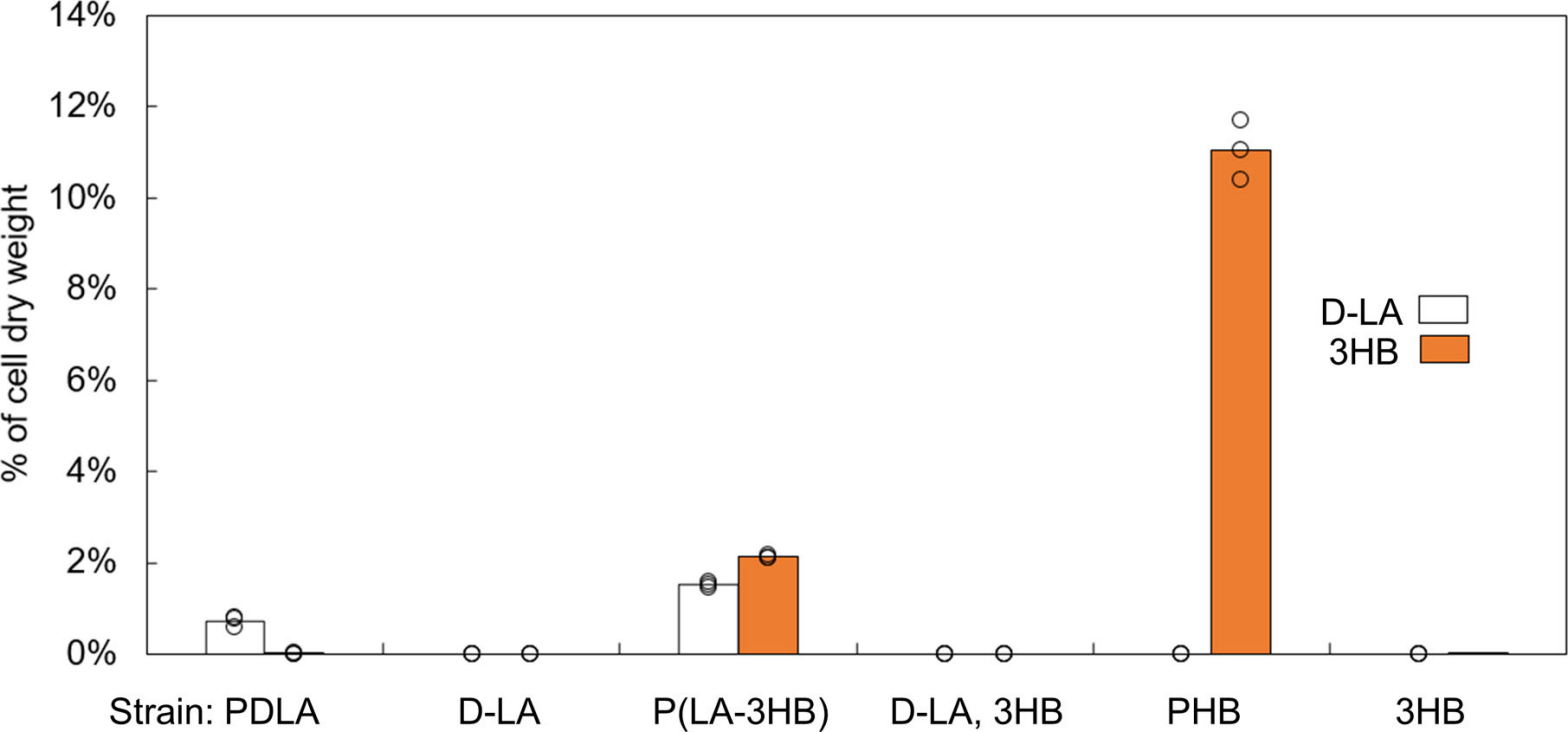
Monomer composition of *S. cerevisiae* strains [% cell dry weight (CDW) of lyophilized cells]. Strains: PDLA: strain producing poly(D-lactic acid) (H5520), D-LA: strain producing D-lactic acid (H5513); P(LA-3HB): strain producing copolymer of D-lactic and 3-hydroxybutyric acids (H5523), D-LA and 3HB-CoA: strain with D-lactic acid and 3-hydroxybutyryl-CoA pathways (H5514), PHB: strain producing poly(hydroxybutyrate) (H5529), 3HB-CoA: strain with 3-hydroxybutyryl-CoA pathway (H5531). Data points from each replicate are marked with circles.

GC–MS methanolysis method was used to degrade the polymer structures and measure the monomer compositions and quantities in relation to the CDW. When cultivation started from OD_600_ 5, the PDLA strain (H5520) contained 0.73 ± 0.12% D-LA of CDW and the P(LA-3HB) copolymer strain (H5523) 1.52 ± 0.06% D-LA and 2.13 ± 0.04% 3-HB of CDW at 72 hr. Total copolymer fraction of the CDW was 3.65%. The PHB strain (H5529) contained 11.05 ± 0.64% 3HB of CDW (Fig. 4). Estimated biomass concentration of strain H5529 at 72 hr was approximately 3.4 g l^–1^ and volumetric PHB yield 380 mg l^–1^. When PDLA and P(LA-3HB) strains were grown starting from OD_600_ 0.2, the PDLA and P(LA-3HB) polymer fractions of the total CDW were 0.63% and 2.54%, respectively (Fig. 3). P(LA-3HB) contained 65% D-LA-monomers and 35% 3HB monomers. The final biomasses at 72 hr were 1.48 ± 0.09 and 1.15 ± 0.05 g l^–1^ for PDLA and copolymer strains, which yields in final polymer concentrations of 9.4 and 29 mg l^–1^, respectively. Control strain producing extracellular D-LA, or D-LA and 3HB monomers (H5513 and H5514) did not contain any D-LA or 3-HB in the GC–MS analysis of cell biomass. Two replicates of control strain carrying only 3HB-CoA pathway (H5531) contained 0.028% and 0.024% 3-hydroxybutyric acid of CDW, while the third replicate did not contain any 3HB.

### Analysis of Polymer Properties

**Fig. 5.**
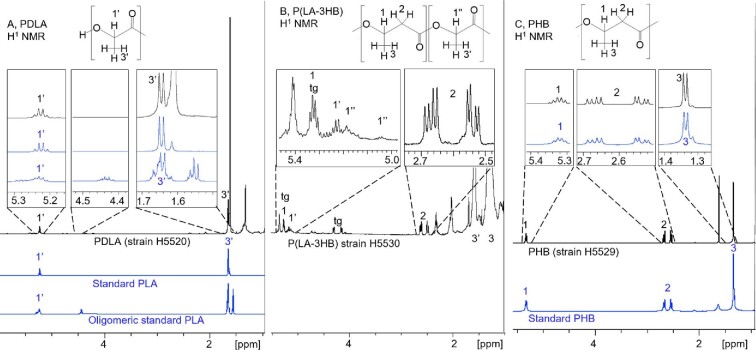
^1^H-NMR spectra of polymers extracted from *S. cerevisiae*. (A) Poly(D-lactic acid) (PDLA) from strain H5520, PLLA standard (95468-1G-F, Sigma-Aldrich, Mw 259 kDa, Mn 103 kDa) and oligomeric PLA standard produced by acidic hydrolysis of commercial PLA (Ingeo 3251D, NatureWorks) containing dimer, trimer, tetramer, and pentamer units. (B) Copolymer P(LA-3HB) from strain H5523. (C) Poly(hydroxybutyrate) (PHB) from strain H5529, PHB standard (363502, Sigma-Aldrich) tg: CH signal of the glycerol backbone of triglycerides.

**Fig. 6.**
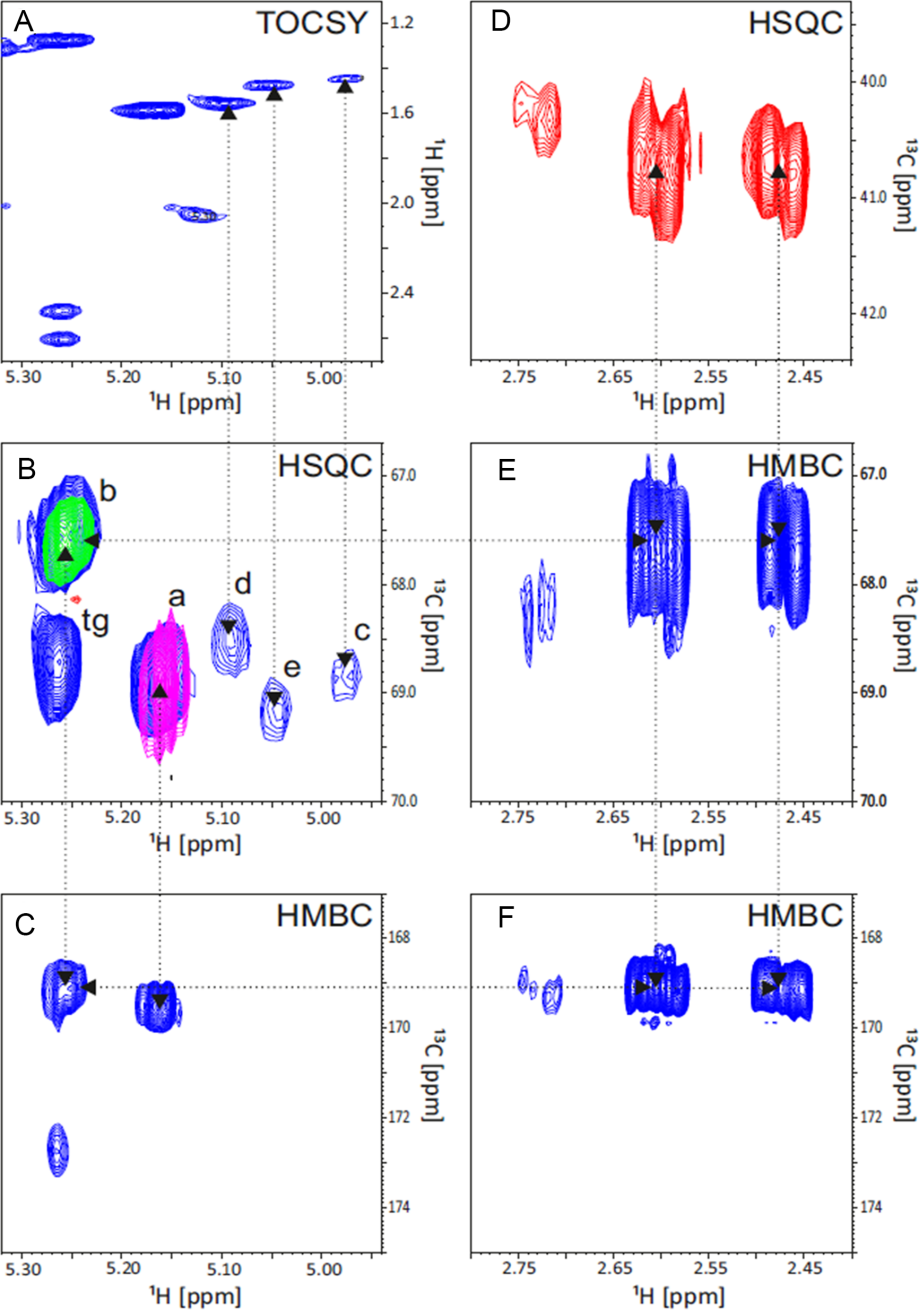
Expansions of 2D total correlation spectroscopy (TOCSY), heteronuclear single quantum correlation (HSQC), and heteronuclear multiple bond correlation (HMBC) spectra of the copolymer P(LA-3HB). (A) Part of the methyl, and CH_2_ region of TOCSY showing the lactate type methyl correlations to the small CH signals observed in HSQC (panel B) and 3-hydroxybutyryl (3HB) CH_2_ and methyl groups. (B) HSQC CH signals of the polymers. Violet and green contours are overlaid spectra of the homopolymers poly(D-lactic acid) (PDLA) (a) and poly(hydroxybutyrate) (PHB) (b), respectively. Three small slightly shifted PDLA type signals (c–e) with TOCSY correlations to methyl regions (panel A) are also observed. CH signal of the glycerol backbone of triglycerides (tg) is possibly overlapping with some of the 3HB signals. (C) HMBC correlations of PDLA and PHB CH protons to CO carbons. (D) HSQC CH_2_ signals of 3HB blocks showing also a small slightly shifted doublet. (E) HMBC correlations of the CH_2_ protons of 3HB blocks to the CH carbon of PHB. (F) HMBC correlations of PHB CH_2_ protons to a CO carbon.

Polymers were extracted from cells and analyzed with GC–MS, SEC, and NMR in order to verify their monomer compositions and structures. The NMR spectra are shown in Figs 5 and 6, and the monomer composition results are summarized in Table 3. The NMR results in Table 3 are estimations of the signals from extracted P(LA-3HB), since their precise integration was not possible due to the low concentration of polymers and background signal from impurities.

**Table 3.** Monomer Composition (mol%) of D-Lactic Acid and 3-Hydroxybutyric Acid (D-LA:3HB) in Polymer Samples

Analysis	PDLA	P(LA-3HB)			PHB	PHB standard
GC–MS from lyophilized cells	100:0	46:55	46:54	45:55	0:100	0:100
GC–MS from extracted polymer	100:0	34:66	55:45	46:54	0:100	0:100
NMR from extracted polymer	100:0	20:80	40:60	40:60	0:100	0:100

*Note*. Samples: PDLA: poly(D-lactic acid) from strain H5520, P(LA-3HB): three replicates of copolymer of lactic and 3-hydroxybutyric acids from strain H5523, PHB: poly(hydroxybutyrate) from strain H5529, and PHB standard from Sigma (Product 363502). Molecular fractions are analyzed with GC–MS from lyophilized cells and from extracted polymers, and with NMR from extracted polymers.

The ^1^H NMR peaks of commercial PLA and PHB polymers are shown as references in the ^1^H NMR spectra of PDLA and PHB polymers produced by yeast (Fig. 5A and C) to confirm the production of PDLA and PHB. An oligomeric PLA sample produced by acidic hydrolysis of commercial PLA (Ingeo 3251D, NatureWorks) containing dimer, trimer, tetramer, and pentamer units (Anghelescu-Hakala & Nyyssölä, 2020) was used for end group analysis. The sample was analyzed by ^1^H NMR to identify the location of the CH groups linked to the end hydroxyl group in the NMR spectra. The CH signals of the hydroxyl terminal were detected at 4.45 ppm in the oligomeric standard sample. Corresponding 4.45 ppm peaks from the PDLA produced by *S. cerevisiae* strains were just above the detection limit and much smaller than in the commercial PLA sample, indicating that the degree of polymerization of yeast PDLA sample was higher than that of the commercial PLA. Due to the low intensity of the end group signal from our sample, the accurate quantification was not possible. In addition, in the oligomeric PLA sample, scattering of the spectra was seen in 5.2 and 1.6 ppm signals due to proximity of polymer ends. Similar scattering was not observed in PDLA produced in yeast nor in the commercial PLLA standard (95468-1G–F, Sigma-Aldrich with weight average molecular weight, Mw, of 259 kDa and number average molecular weight, Mn, of 103 kDa). The ^1^H NMR spectrum of P(LA-3HB) copolymer shows, in addition to the signals seen in the PDLA and PHB spectra, some slightly shifted signals (1″ in Fig. 5B). The CH proton signals of 3HB units were severely overlapped by the signal of the middle proton of glycerol backbone of triglyceride contaminants in extracted polymer samples (tg). Also, the methyl signals were overlapped with the strong background signals. Therefore, the copolymer structures were analyzed also by 2D NMR. Fig. 6B shows HSQC CH signals of P(LA-3HB) in blue and the overlaid spectra with PDLA and PHB in violet and green, respectively. The triad LA-LA-LA signal, (a) in Fig. 6B, is identical to the homopolymer PDLA (violet signal) and triad signal 3H-3HB-3HB (b) is identical to PHB (green). In addition to the homopolymer CH signals, small signals with slightly different chemical shifts were observed (Fig. 6B): (d), (e), and (f). These signals were not present in the homopolymer spectra and thus indicate copolymeric structures. According to the TOCSY spectrum, these signals belong to lactate type spin systems (Fig. 6A). They were assigned to central lactate units in triad sequences (c) 3HB-LA-3HB, (d) and (e) either 3HB-LA-LA or LA-LA-3HB (Yamada et al., 2009). Integration of these LA HSQC signals gives approximate relative populations of the four structures: a (LA-LA-LA): 79%, c (3HB-LA-3HB): 5%, d or e (3HB-LA-LA or LA-LA-3HB): 16%. The HSQC also shows additional CH signals for 3HB units, but due to the overlapping with triglyceride signal (tg) their interpretation is not possible. Likewise, 3HB CH_2_ HSQC area also shows additional slightly shifted signals of the copolymer (Fig. 6D) having HMBC correlations to the 3HB CH signals (Fig. 6E). Both LA and 3HB homopolymer type CH protons also have HMBC correlation to the CO area at 169.55 and 169.11 ppm, respectively (Fig. 6C). However, due the low amounts of copolymeric structures, similar correlations could not be observed for the copolymeric CH protons.

SEC results are summarized in Table 4 and SEC chromatograms are presented in Fig. 7. Successful SEC results from PDLA and P(LA-3HB) samples were obtained when extracted polymers were washed both with methanol and diethylether. Commercial PHB standard (363502, Sigma) gave over fivefold higher polydispersity with HFIP-based SEC system in comparison to chloroform-based system. Commercial PHB showed poor solubility in both solvents at room temperature. Solid particles were visible in the HFIP solvent even after dissolving the sample for 3 days with mixing. Heating of commercial PHB in chloroform solvent, in addition to mixing at room temperature for 7 days, resulted in clear solution. Similar solids particles were not visible when yeast PHB was dissolved in HFIP and when yeast PDLA and copolymer were washed with diethyl ether before chloroform-based SEC analysis. Melting temperature of the PHB samples was 176.7 ± 0.57ºC. Melting temperature of a commercial PHB standard was 174.5ºC, measured from one sample. The DSC thermograms are presented in Fig. 8.

**Table 4.** Molecular Weights of PDLA, P(LA-3HB), and PHB Polymers

Sample	SEC system	Mn (Da)	Mw (Da)	PDI
PDLA	Chloroform	5,290	6,250	1.18
P(LA-3HB)	Chloroform	19,150	24,640	1.29
PHB (replicate 1)	HFIP	336,170	1,089,630	3.24
PHB (replicate 2)	HFIP	336,650	1,193,400	3.54
PHB (replicate 3)	HFIP	334,900	1,108,270	3.31
Commercial PHB	HFIP	128,510	1,592,100	12.39
Commercial PHB	Chloroform	258,200	563,400	2.18

*Note*. Mw, weight average molecular weight; Mn, number average molecular weight, PDI, polydispersity.

## Discussion

Polyhydroxyalkanoates are interesting family of microbially made biodegradable polymers. Their structures are defined by the availability of suitable monomers and substrate specificity of the PHA synthase enzyme. In this study, we demonstrated for the first time that robust yeast *S. cerevisiae* can polymerize D-lactic acid alone, or together with 3-hydroxybutyrate, when PHA synthases with a broad substrate specificity are expressed. The engineered strains produced P(LA-3HB) and PDLA from glucose with accumulation of 3.6% and 0.73% of CDW, and with weight average molecular weights of 24.6 and 6.3 kDa, respectively. The key enzymes, PHA synthases, PhaC1Pre and PhaC1437_Ps6–19_, are reported to polymerize also other 2-hydroxyacyl-CoAs, 3-hydroxylacyl-CoAs, monomers with aromatic side chains and mcl monomers (Choi et al., 2016; Jae et al., 2013; Solaiman, 2003; Yang et al., 2011, 2018). Functionality of these wide specificity enzymes in *S. cerevisiae* enables future tailoring of polymer properties in yeast.

The D-lactic acid fraction in the copolymer P(LA-3HB) produced in this study varied from 46 to 65 mol %. Similar, approximately 50% lactic acid fraction was reported also when PhaC1437_Ps6–19_ was expressed in recombinant *E. coli* (Yang et al., 2011). P(LA-3HB) copolymers with these D-lactic acid fractions have been reported to have lower melting temperatures and higher elasticities than those of PDLA and PHB (Taguchi et al., 2008; Yamada et al., 2009, 2011). According to our 2D NMR results of P(LA-3HB), the D-LA and 3HB monomers are covalently attached forming copolymers with random structure. Majority of D-LA monomers, 79%, were located between two other D-LA monomers, 16% between D-LA and 3HB monomers, and only 5% between two 3HB monomers. The result indicates that P(LA-3HB) copolymer produced in this study contained D-LA repeating sequences. Repeating D-LA sequences in P(LA-3HB) copolymers have also been found in some bacterial studies (Ochi et al., 2013; Tajima et al., 2012; Yamada et al., 2009). The repeating monomer sequences are interesting since they might enable crystallization of otherwise amorphous polymer and thus influence its thermal properties (Peacock & Calhoun, 2006). According to SEC analysis, the P(LA-3HB) polymer had molecular weights, Mn and Mw, of at least 19.2 and 24.6 kDa, respectively. These molecular weights are slightly smaller than molecular weights, Mn 22 kDa and Mw 39 kDa, reported for P(LA-3HB) with similar lactate fraction and produced by *E. coli* expressing the same PHA synthase PhaC1437_Ps6–19_ (Yang et al., 2010). The relatively low molecular weights, in comparison to molecular weight of PHB, could result from the high lactic acid fraction of 46–65 mol%. High lactic acid fractions in P(LA-3HB) copolymer have corresponded to low molecular weights of the polymer. Reasons for low molecular weights are discussed below also for PDLA. The SEC result from our study confirms the successful polymer production, isolation, and purification. Unimodal distribution of molar masses and narrow polydispersity (PDI) indicate that purified polymers are free of small molecular weight contaminants. These shorter polymers could be suitable for applications like aqueous dispersion coatings for packaging materials.

PDLA and P(LA-3HB) contents in this study, 0.73% and 3.6%, are similar to the lower range reported in bacterial hosts, the whole range varying from 0.5% to 75% (Jung et al., 2010; Kadoya et al., 2015; Yang et al., 2011). Yeast strains produced approximately 9 g l^–1^ extracellular D-LA indicating that the D-LA production rate exceeded the capability of cells to polymerize it. In addition, the PDLA and P(LA-3HB) producing strains grew remarkably slower compared to their control strains without the Pct and PhaC enzymes. Possibly the use of acetyl-CoA for generation of D-lactyl-CoA affected their growth. Wild type Pct from *C. propionicum* has been reported to inhibit cell growth in *E. coli* (Selmer et al., 2003). Further optimization of ldhA, Pct, and PHA synthase expression levels and activities could thus improve cell growth, increase the polymer yield, and prevent excessive extracellular D-lactic acid accumulation. For example, in the recombinant *Corynebacterium glutamicum* PDLA yield has been increased fourfold by enhancing the PhaC1PsSTQK PHA synthase activity (Matsumoto et al., 2014).

The PLA content of the CDW in this study was 10-fold lower compared to that of PHB. This may be due to non-optimal availability of D-lactyl-CoA as a precursor, lower activity of the engineered synthase enzymes towards D-lactyl-CoA, compared to 3-hydroxy butyryl-CoA, or physical properties of the formed polymer, as discussed below. Similar decrease has been observed with engineered *E. coli* strains where the highest reported PHB yields (Aramvash et al., 2015; Zhang et al., 2018) have been 10-fold higher than the highest obtained PDLA yields (Jung et al., 2010; Yang et al., 2011). It has been hypothesized that D-lactyl-CoA could occasionally terminate the growth of the polymer chain (Yang et al., 2011). Recent *in vitro* study, with PhaC1PsSTQK PHA synthase (carrying two amino acid changes, S325 and Q481), reports termination of PDLA polymerization when PDLA chains reach molecular weight of 3 kDa (Matsumoto et al., 2018). The reason for this relatively short molecular weight was suggested to be the high glass transition temperature of PDLA homopolymers (60°C) that would decrease polymer mobility notably at common reaction temperatures of 30–37°C. However, *in vivo* studies using PHA synthases with three or four active mutations in their active sites report production of 4- to 18-fold longer polymers. Following molecular weights of PDLA are reported in *E. coli*: 23 kDa (Mn) and 55 kDa (Mw) with PhaC1Pre carrying amino acid changes E130, S325, S477, and Q481 (Yang et al., 2011) and 30 kDa (Mw) with PhaC1310_Ps6–19_ carrying amino acid changes E130D, S477F, and Q481K (Jung et al., 2010). In addition, molecular weights of 12.5 (Mn) and 50.5 (Mw) are reported in yeast *Y. lipolytica* expressing PHA synthase from *Pseudomonas aeruginosa* with amino acid changes E130D, S325T, S477R, and Q481M (Lajus et al., 2020). In the present study, SEC analysis confirmed that for PDLA produced in *S. cerevisiae* molecular weights, Mw and Mn, are at least 6.3 and 5.3 kDa, respectively.

The PHB yield of 11% of CDW in this study is comparable to other studies of PHB production in *S. cerevisiae* (Breuer et al., 2002; Carlson & Srienc, 2006). However, optimization of pathway enzymes, carbon sources, aeration, and redox balances in the cell can increase the polymer yield in *S. cerevisiae* up to 16.4 % (de Las Heras et al., 2016; Kocharin et al., 2012; Portugal-Nunes et al., 2017). The highest reported PHA percentages per CDW in yeast are 30% of PHB for *P. pastoris* (Vijayasankaran et al., 2005) and 30% of mcl PHAs for *Y. lipolytica* (Rigouin et al., 2019). These values were measured as averages of the cell population. However, Carlson & Scrienc (2006) discuss, based on fluorescence microscopy images, that some *S. cerevisiae* cells could have accumulated up to 50% PHB of CDW while others seemed empty. Population heterogeneity was seen also in fluorescence based flow cytometry analysis of PHB producing *S. cerevisiae* (Kacmar et al., 2006). These observations suggest that PHA production in yeast can still be increased both at individual cell level and by reducing the population heterogeneity.

The suitability of *S. cerevisiae* for PHA production is also supported by SEC results of the extracted PHB in this study. The average molecular weights, 336 kDa (Mn) and 1130 kDa (Mw), of PHB in this study are higher than average molecular weights, 130 kDa (Mn) and 200 kDa (Mw), of PHB produced from acetate by *Y. lipolytica* expressing the same PHA synthase from *C. necator* (Li et al., 2016). In fact, molecular weights in this study are similar to the reported molecular weights of PHB produced with the native bacterial producer *C. necator*, 400 kDa (Mn) and 1136 kDa (Mw) (Meixner et al., 2018), and 1400 kDa (Mw) (Fei et al., 2016). The melting temperature of the PHB produced by *S. cerevisiae*, 176.0°C, is also in accordance with results from literature for bacterial PHB (Lee, 1996). These results indicate that recombinant *S. cerevisiae* strains can produce polymers with properties comparable to those of the polymers produced by the engineered or native PHA producing bacteria.

The *S. cerevisiae* platform can be further developed for production of PHA polymers in non-buffered or low pH conditions (Fernandes et al., 2005; Mitsui et al., 2020; Rajkumar et al., 2016; Zhong et al. 2021), and for utilization of various industrial side streams as raw materials. In addition, the use of wide substrate specificity of the studied PHA synthases enables tailoring of the polymer properties in future.

## Abbreviations

ldhA, stereospecific D-lactate dehydrogenase; Pct, propionyl-CoA transferase; PDLA, poly(D-lactic acid); PHA, poly(hydroxyalkanoate); PhaA, acetyltransferase from *Cupriavidus necator*; PHB, poly(hydroxybutyrate); PhaB1, acetoacetyl-CoA reductase from *C. necator*; PhaC1, poly(hydroxyalkanoate) synthase from *C. necator*; P(LA-3HB) copolymer of D-lactic and 3-hydroxybutyric acids

## Data Availability

The datasets used and analyzed during the current study are available from the corresponding author on reasonable request.
